# C/EBPδ Gene Targets in Human Keratinocytes

**DOI:** 10.1371/journal.pone.0013789

**Published:** 2010-11-02

**Authors:** Serena Borrelli, Daniele Fanoni, Diletta Dolfini, Daniela Alotto, Maria Ravo, Olì Maria Victoria Grober, Alessandro Weisz, Carlotta Castagnoli, Emilio Berti, M. Alessandra Vigano, Roberto Mantovani

**Affiliations:** 1 Dipartimento di Scienze Biomolecolari e Biotecnologie, Università degli Studi di Milano, Milano, Italy; 2 Istituto di Scienze Dermatologiche, IRCCS Fondazione Ospedale Maggiore Policlinico, Mangiagalli e Regina Elena, Università degli Studi di Milano, Milano, Italy; 3 Dipartimento di Chirurgia Plastica - Banca della Cute, Ospedale CTO, Torino, Italy; 4 Dipartimento di Patologia Generale and Centro Grandi Apparecchiature, Seconda Università di Napoli, Napoli, Italy; 5 AIRC Naples Oncogenomics Centre, c/o CEINGE Biotecnologie Avanzate, Napoli, Italy; 6 Università di Milano-Bicocca, Milano, Italy; Institute of Genetics and Molecular and Cellular Biology, France

## Abstract

C/EBPs are a family of B-Zip transcription factors -TFs- involved in the regulation of differentiation in several tissues. The two most studied members -C/EBPα and C/EBPβ- play important roles in skin homeostasis and their ablation reveals cells with stem cells signatures. Much less is known about C/EBPδ which is highly expressed in the granular layer of interfollicular epidermis and is a direct target of p63, the master regular of multilayered epithelia. We identified C/EBPδ target genes in human primary keratinocytes by ChIP on chip and profiling of cells functionally inactivated with siRNA. Categorization suggests a role in differentiation and control of cell-cycle, particularly of G2/M genes. Among positively controlled targets are numerous genes involved in barrier function. Functional inactivation of C/EBPδ as well as overexpressions of two TF targets -MafB and SOX2- affect expression of markers of keratinocyte differentiation. We performed IHC on skin tumor tissue arrays: expression of C/EBPδ is lost in Basal Cell Carcinomas, but a majority of Squamous Cell Carcinomas showed elevated levels of the protein. Our data indicate that C/EBPδ plays a role in late stages of keratinocyte differentiation.

## Introduction

The skin is a multilayered epithelium that enables organisms to be protected from the exterior, while keeping homeostasis of fluids. Skin differentiation is a lifelong process that leads to the expansion of cells with specific, exquisite features from a relatively few progenitors that guarantee the maintenance of the stem cells pool. Renewal of stem cells and ongoing active terminal differentiation require the progressive fine tuning of transcriptional programs, which are masterminded by Transcription Factors (TFs). Several TFs have a role in keratinocytes physiology. Specifically, genetic experiments established that p63, IRF-6, KLF4, Gata3 and C/EBPs are important [1].

C/EBPs are a family of six B-Zip TFs that activate and repress transcription of genes involved in differentiation and growth control [2]. The two most studied members of the family, C/EBPα and C/EBPβ are required for differentiation of adipocytes and hematopoietic cells [3]–[6]. Inactivation of C/EBPε leads to lack of natural killer cells [7]. C/EBPζ -CHOP, DDIT3- is involved in the response to noxious signals, such as ER-stress and DNA-damage [8]. C/EBPδ KO mice have a mammary phenotype, with an alteration in the involution of the mammary glands upon lactation [9], [10]. Similarly to C/EBPβ adipocyte differentiation is impaired in cells that lack C/EBPδ when cultured *in vitro* [6].

In the skin, C/EBPα is expressed mostly in the upper layers [10] and mice with selective ablation of C/EBPα in the skin have no apparent phenotype in normal conditions, but are subject to increased RAS-mediated tumorigenesis [11]. C/EBPβ is expressed in the cytoplasm of basal keratinocytes [10] and in the nuclei of the spinous layer [12]. Mice with selective ablation of C/EBPβ in the skin have epidermal hyperplasia with downplay of Keratin 1 and 10 expression [13]. Contrary to C/EBPα, C/EBPβ ablation in the skin suppresses RAS-mediated tumorigenesis [14]–[17]. Importantly, the combined ablation of C/EBPα and C/EBPβ leads to profound alteration in interfollicular epidermis due to increased proliferation of basal cells, defective differentiation and insufficient barrier function [18]. These mice show suprabasal p63 expression. Vinson's group reported on a transgenic model expressing a dominant negative C/EBP –termed A-C/EBP- in basal keratinocytes: these mice have hyperplasia of the basal epidermis and increased apoptosis of the upper layers [19]: p53 and pro-apoptotic markers are induced and C/EBPβ dramatically reduced. A-C/EBP is not C/EBPβ-specific, thus C/EBPδ might contribute to the observed phenotype. Although C/EBPδ KO mice have no apparent skin alterations [20], expression is high in human interfollicular skin, and negatively regulated by p63 [21]. Importantly, in the multi-layered epithelia of the corneal limbus, C/EBPδ is upstream of p63 and thought to be a marker of stemness [22].

Recent reports reveal alterations in the structure or expression levels of C/EBP family members in a variety of human cancers. For example, down-regulation of C/EBPα is seen in several human malignancies, including skin [23], whereas C/EBPβ is overexpressed in carcinogen-induced skin tumors [24] and breast cancer [25]. Moreover, “loss of function” alterations of CEBPδ and promoter methylation of the C/EBPD *locus* have been observed in primary human breast tumors [26], [27]. Through ChIP on chip experiments, C/EBPα and C/EBPβ targets were identified in 3T3-L1 adipocytes [28], and C/EBPδ in mammary cells undergoing growth arrest [29]. To reconstruct the C/EBPδ network in keratinocytes, we identified targets, through the use of RNAi inactivation coupled to gene expression profiling, and ChIP on chip technology.

## Materials and Methods

### Cells and culture conditions

First passage human primary keratinocytes were derived from breast of healthy individuals undergoing plastic surgery and grown on a feeder-layer of lethally irradiated 3T3 cells in DMEM F12 added of Insulin (5 µg/ml), EGF-R (10 ng/ml), hydrocortisone (0.4 µg/ml), T3 (2 nM), Cholera toxin (0.1 nM) and transferrin (5 µg/ml). The preparation of primary keratinocytes was performed at the“Banca della Cute”-CTO Hospital in Torino (I), and the procedure was approved by the Ethics Commettee “Comitato Etico Interaziendale S. Giovanni Battista CTO-M.Adelaide”. Informed verbal consent from the patients was obtained as stated in procedure, for which approval was granted by the Ethics Committee.

### RT-PCR and transfections

For RNA profilings, 10^6^ first passage primary keratinocytes from healthy individuals were transfected with Nucleofector (Amaxa, D) according to the Manufacturer' conditions with the off-target control siRNA oligos (Sigma), or with an oligonucleotide targeting human C/EBPδ GACUCAGCAACGACCCATuu [21]. The mRNAs were extracted 48 hours after transfections with the RNA-Easy kit (Qiagen, D). For cDNA synthesis, 2 µg of RNA were used with M-MLV-RT kit (Invitrogen, USA). Semi-quantitative PCR analysis o the C/EBPδ mRNA were performed with specific primers (AGTTCTTGGGACATAGGAGCGCA; GTACCTTAGCTGCATCAACAGGAG). qRT-PCR analysis was used to validate the profiling arrays, with a Biorad MyIQ single colour thermal cycler and a SYBR Green PCR Master mix. Specificity of products was monitored with a heat dissociation curve, fold enrichment was calculated with the formula 2^−ΔCt^ where the Ct represented the threshold cycles of the input, the specific antibody and the negative antibody; a further normalization with the enrichment obtained on a negative genomic region (Centromeric Satellite 11) was applied. A list of the RT-PCR primers is provided in Table S1. Transfections with 4 µg of Sox2 and MafB expression vectors, or empty pcDNA3 control, were used; cells were split in two after 24 hours, and one of the culture was differentiated by addition of 1,4 mM CaCl_2_.

### ChIP on chip

ChIP analysis was carried out with the method described in Ref. 21 with an anti-C/EBPδ antibody (Active Motif #39006). The immunoprecipitated DNA was analyzed with specific primers in semi-quantitative PCR assays with the primers listed in Table S2.

The generation of amplicons from the individual ChIPs was performed following the protocol of LM-PCR described in Refs. 30, 31. Briefly, two unidirectional linkers were annealed and ligated to the chromatin IPs, previously blunted by T4 DNA polymerase. The first amplicons were generated by PCR (15 cycles). The reaction was purified using the GFX PCR purification kit (Amersham Biosciences) according to the Manufacturer's instructions. A fraction of these initial reactions was used to generate more amplicons for 30 additional cycles. After purification, the DNA was quantified and examined by gene specific PCR to ensure that the initial enrichment was maintained. 5 µg of amplicons for Flag and input DNA (subjected to the same number of PCR manipulations) were labeled using the LabelIT Cy5/Cy3 Nucleic acid labeling kit (Mirus), following the manufacturer's instructions, with a reagent to DNA *ratio* of 2.5 for Cy5 (IPs) and 1.5 for Cy3 (Input). The hybridization and washing conditions for these slides were described previously [30], [31]. The CpG 21K slides were purchased from University Health Network, Toronto, Canada. The hybridized microarrays were scanned and analyzed using a ScanArray 4000 and QuantArray analysis software (Packard). Features of poor intensity (<500) and those which did not meet the quality control criteria (visual inspection, spot circularity, spot uniformity and background uniformity for both channels) were discarded. After the background subtraction for each spot, the data were normalized to median, that is, the *ratio* of the median value of all spots in the Cy5 channel (IP DNA) and the *ratio* of the median value of the control channel (Cy3 Input). The 21K CpG island array was described in Ref. 31. We considered positive all the clones which satisfied the described criteria: >1.5-fold enrichment in the C/EBPδ samples over the negative antibody -Flag- samples in each of the performed experiments. The sequences of the positive CpG islands clones were retrieved from both the Sanger center (http://www.sanger.ac.uk/HGP/cgi.shtm) and the UHN Cancer Center (http://derlab.med.utoronto.ca/CpGIslands.htm) and mapped on the human genome using Blat (http://genome.ucsc.edu). Approximately 2 kb of genomic regions 5′ and 3′ of the CpG islands clones were considered and one or more Gene ID were assigned to each clone, if they laid in these regions. GO enrichment analysis was performed at http://bioinfo.vanderbilt.edu/webgestalt.

### RNA profiling

Keratinocytes from three independent healthy donors were transfected to inactivate C/EBPδ. 2 µg of each sample were pooled, for further processing. For each sample, 500 ng of total RNA were synthesized to biotinylated cRNA using the Illumina RNA Amplification Kit (Ambion, USA). Synthesis was carried out according to the Manufacturers' instructions. From each sample, three technical replicates were produced and 750 ng cRNA were hybridized for 18 hrs to HumanHT12 v. 3.0 Expression BeadChips (Illumina Inc., USA) according to the protocol provided by the manufacturer. Hybridized chips were washed and stained with streptavidin-conjugated Cy3 (GE Healthcare, USA). BeadChips were dried and scanned with an Illumina BeadArray Reader (Illumina, USA). For data analysis, the intensity files were loaded into the Illumina BeadStudio v. 3.1.3.0 software for quality control and gene expression analysis. First, the quantile normalization algorithm was applied on the dataset to correct systematic errors. Background is subtracted. For differential expression analysis, technical replicates of each sample were grouped together and genes with a detection *p*-value below 0.01, corresponding to a false positive rate of 1%, were considered as detected. Differently expressed genes were selected with Diff Score cutoff set at ±30; genes with a differential expression of >1.5 between siC/EBPδ and siScramble were retrieved.

### Western blot and Immunohistochemistry

Western blot analysis was performed using standard procedure with a Pierce secondary antibody and PDS detection system (Genespin, I).

For IHC, tissue samples were fixed in buffered formalin, dehydrated, embedded in paraffin wax and sectioned. After deparaffinizing and rehydrating, each tissue section was immersed in citrate buffer 0.01M pH 6 or EDTA 0.05M pH 8 or Tris-HCl 0.05M pH 9.9, boiled 3 times for 5 minutes in a pressure cooker and washed with TBS buffer. Each section was placed on the Dako cytomation automated immunostainer and incubated with the specific monoclonal antibody at room temperature for 45 minutes, washed with TBS pH 7.6 and incubated in a biotinylated goat anti-mouse anti-rabbit immunoglobulins (Dako REAL™, Dako, Dn) at room temperature for 30 minutes. After incubation with the secondary antibody and a new wash with TBS pH 7.6, sections were incubated with streptavidin conjugated to alkaline phosphatase at room temperature for 30′. A red chromogen solution was prepared as indicated by Dako REAL™ datasheet. Each section was counterstained in Mayer's Hematoxylin solution and coverslipped. Skin cancer tissue array included 48 cases of normal, benign and cancerous tissue of the skin and subcutaneous tissues in duplicates. All the tissues were from surgical resection. They were fixed in 10% neutral buffered formalin for 24 hours and processed using identical SOPs. Sections were picked onto Superfrost Plus or Apes coated Superfrost slides. (Skin Tumor Tissue Array, BioChain, cat. Z7020093). Anti-C/EBPδ antibody (Active Motif, USA) was used in this analysis, together with the 4A4 anti-p63 as a control.

## Results

### Identification of C/EBPδ *loci* in human keratinocytes

**Figure 1:**
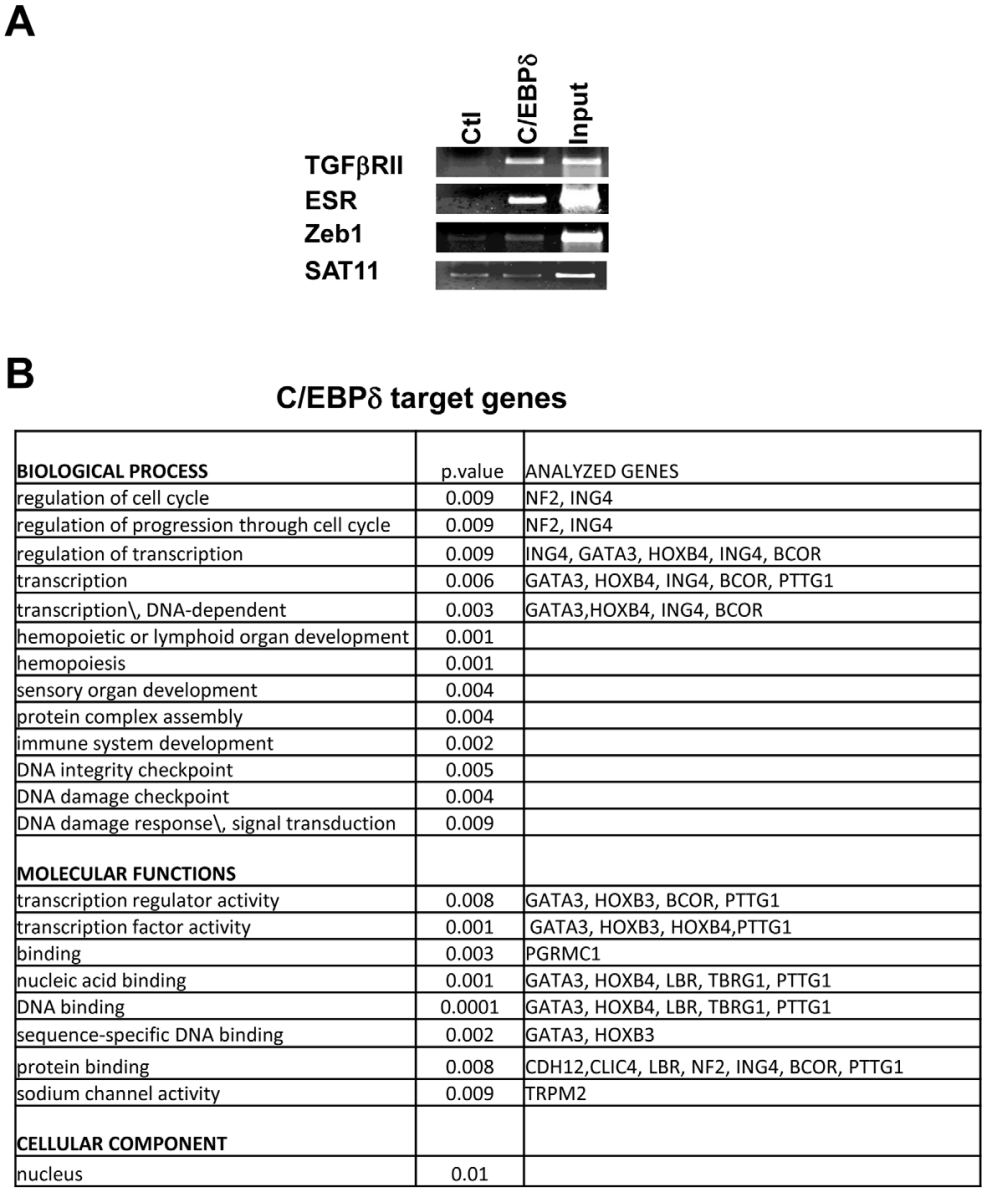
Identification of C/EBPδ targets by ChIP on chip. **A.** Generation of the ChIP amplicons for ChIP on chip experiments. ChIP of human primary keratinocytes with C/EBPδ and control antibodies. The known targets tested are indicated. The normalization of the immunoprecipitated material was performed by evaluating a negative genomic region (Centromeric Satellite 11). **B.** Gene Ontology analysis of C/EBPδ targets genes from the ChIP on chip screening.

**Figure 2:**
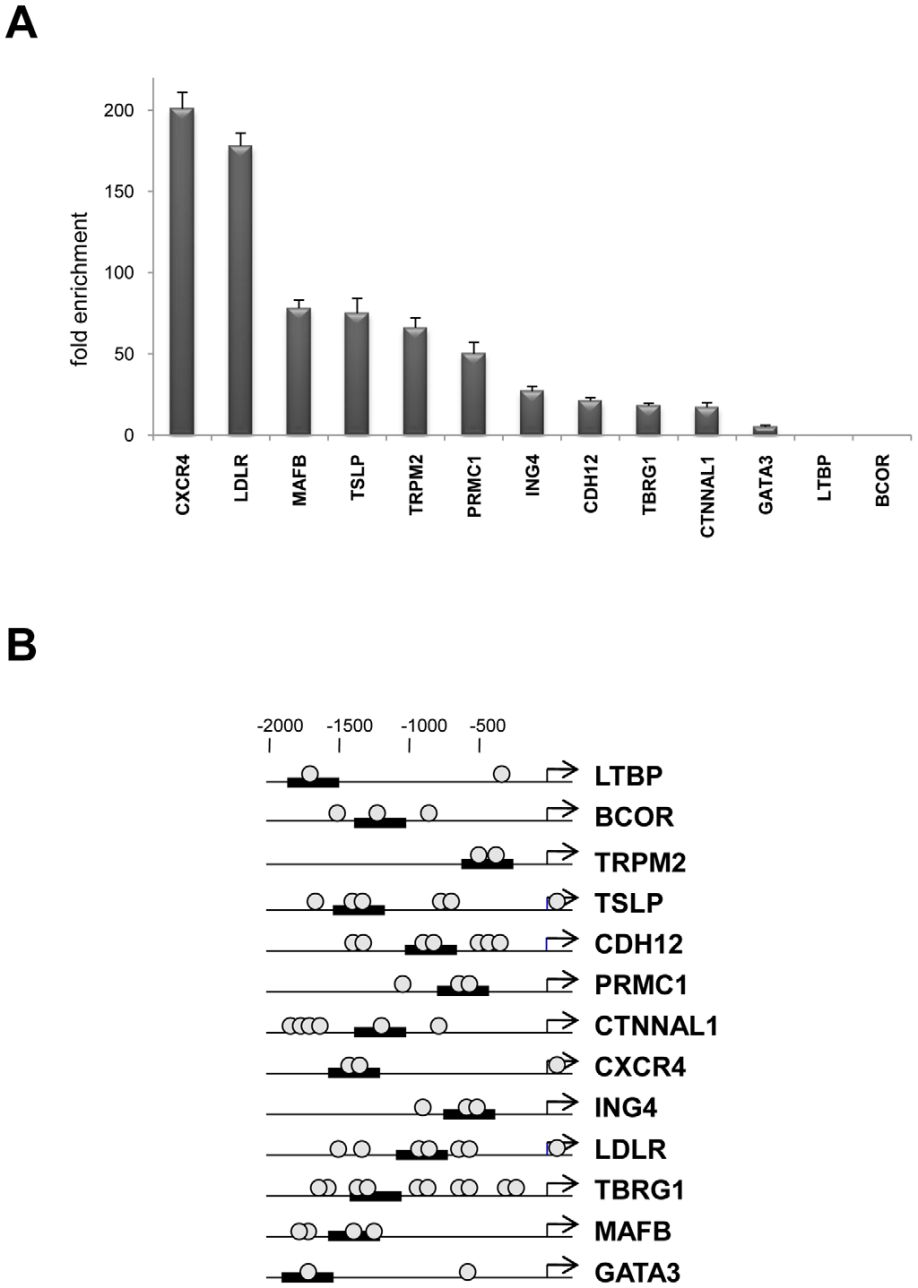
Validation of C/EBPδ targets in primary keratinocytes. **A.** Analysis of the *loci* targeted derived from the ChIP on chip experiments by qPCR analysis of ChIPs with anti-C/EBPδ and control antibodies from human primary keratinocytes. **B**. The position of the ampolicons (black bars) and of the putative C/EBP consensus site (circle) are indicated for targets analyzed in A.

C/EBPδ emerged in expression profilings of human HaCaT and primary keratinocytes in which p63 was functionally inactivated by RNAi [21], [32]. To identify C/EBPδ targets *in vivo*, we performed ChIP on chip analysis with a specific antibody on chromatin derived from keratinocytes of three healthy individuals undergoing plastic surgery. The enrichment over the Flag control was first tested on three promoters previously identified as *bona fide* C/EBPδ targets [21]: Figure 1A shows that TGFβII-R, ESR and Zeb1 promoters, unlike satellite DNA, are specifically enriched in the C/EBPδ IP with respect to control IP,. We hybridized DNA immunoprecipitated with C/EBPδ and Flag control antibodies to the 21K CpG islands array, together with DNAs from Input controls. With a *ratio* of 1.5 enrichment over Inputs, 136 C/EBPδ *loci* were identified. The sequences of these CpG islands spots were retrieved and mapped on the human genome (Table S3). For validation of the ChIP on chip, we selected 13 *loci* near gene promoters and located potential C/EBP binding sites by rVista and ConSite [33], [34] with JASPAR. Amplicons were designed in the area and ChIPs from independent human primary keratinocytes performed. A schematic representation of the selected promoters, with the position of the predicted C/EBP sites and the results of the ChIP experiments are shown in Fig. 2. Ten *loci* were enriched, one –Gata3- was modestly enriched, and LTBP and BCOR did not show credible binding, at least in the region we analyzed. It should be noted that this analysis is complex, as the bound site(s) could be within 0.5 Kb on either side of the CpG island clone identified.

Functional mining of the associated genes was performed by GO analysis and a few terms emerged with a good degree of robustness: *cell cycle*, *transcription* and *DNA-damage checkpoint* in the Biological Process category; *transcription factor* and *DNA-binding* in Molecular Function, and *nucleus* in the Cellular Component category (Fig. 1B). Comparison with ChIP on chip experiments performed in mammary cells upon overexpression of C/EBPδ [29] yielded no overlap. The difference in the arrays -12K vs 21K arrays used in our study- the relative paucity of the targets identified (100 vs 136) and the difference of the cellular context –the MCF12A mammary cell line transfected with C/EBPδ vs normal keratinocytes- might account for this finding. Nevertheless, many GO enriched categories were similar: *cell adhesion*, *cell cycle*, *transcription*, *DNA-damage* [29].

### Identification of C/EBPδ-regulated genes in human primary keratinocytes

**Figure 3:**
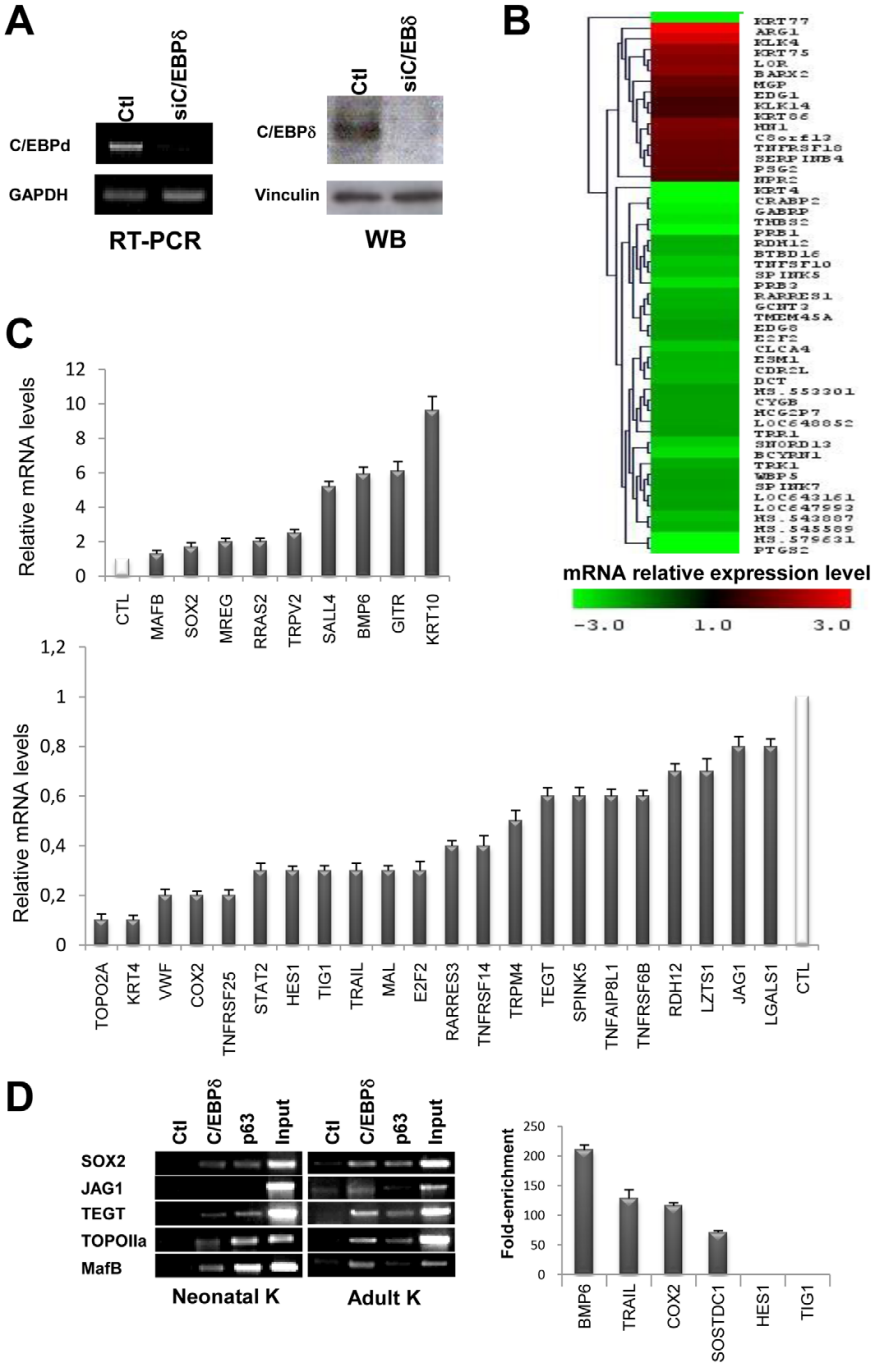
Identification of C/EBPδ-regulated genes in human keratinocytes. **A**. Left Panels, RT-PCR analysis of human primary keratinocytes after C/EBPδ RNAi inactivation at 48 hours post-transfection. cDNA normalization was performed with GAPDH. Right Panels, Western Blot analysis of the same C/EBPδ-inactivated human primary keratinocytes with the C/EBPδ antibody. Vinculin was used as a loading control. **B**. Heat map showing the mRNA expression levels of several classes of genes after C/EBPδ inactivation. **C**. qRT-PCR analysis of C/EBPδ regulated genes that emerged from the expression profiling. **D**. ChIP analysis of promoter regions of C/EBPδ-regulated genes with chromatin from human primary keratinocytes, with α-C/EBPδ and control antibodies. In the Left Panel, adult and neonatal keratinocytes were analyzed in semi-quantitative PCR. In the Right Panel, the targets were validated by qPCR in adult keratinocytes.

We also sought to identify genes whose expression varies inactivating C/EBPδ in human primary keratinocytes. We RNAi-inactivated C/EBPδ with one of the siRNAs previously tested [21]: the process was efficient, as checked in RT-PCR and Western blot analysis (Fig. 3A). Keratinocytes derived from three healthy individuals undergoing plastic surgery, independent from those used in ChIP on chips, were transfected with control and C/EBPδ siRNAs, followed by gene expression profiling with the Illumina platform, in triplicate. For subsequent analysis, we considered a threshold of 1.5-fold over scramble siRNA samples. Heat maps of these experiments are shown in Fig. 3B: 420 up-regulated and 163 down-regulated genes were identified (Table S4).

We validated by qRT-PCR 22 genes that decrease upon C/EBPδ removal and 9 that increase: Fig. 3C shows that all genes were regulated in the expected way. Variation were quantitatively greater in qRT-PCR compared to the array data, an indication that the threshold considered is relatively stringent and the cohort of truly regulated genes is larger. To ascertain whether the genes are direct targets of ChIPs, we performed ChIPs with chromatin from adult and neonatal primary keratinocytes on five promoters –Sox2, TEGT, MafB, TopoIIα and Jag1- with antibodies against C/EBPδ; p63 was used as a positive control, since these genes are also p63 targets. Fig. 3D (Left Panels) shows that most targets are positive, with the exception of Jag1 in neonatal keratinocytes. In the Right Panels qPCR analysis was performed on additional targets in adult keratinocytes and two targets -TIG1 and Hes1- were not validated in the core promoter region considered. Overall the validation data support the results of the profiling analysis.

We performed functional mining of the C/EBPδ regulated genes by GO analysis (Figure S1). Among the genes whose expression increased after C/EBPδ removal, hence repressed, the *epidermis development*, *transcription* and *cell migration* classes emerged with p values lower than E-05. Among the genes decreased by C/EBPδ removal, therefore normally activated, *cell cycle* and *mitosis* were at the top of the Biological Process list, with *lipid biosynthesis*, *development* (*ectoderm*, *muscle*, *immune system*) having p values lower than E-07. In the Cellular Component category, we found *chromosomal part* and *spindle*, and in the Molecular Function few terms were enriched. In conclusion, this analysis is in good accordance with the terms retrieved from the ChIP on chip experiments, since terms like *cell cycle* and *transcription* predominate. Note, however, that only one gene –MafB- was found in both lists, possibly due to the use of different genomic platforms.

### Comparison between the C/EBPδ and p63 targets in keratinocytes

**Figure 4:**
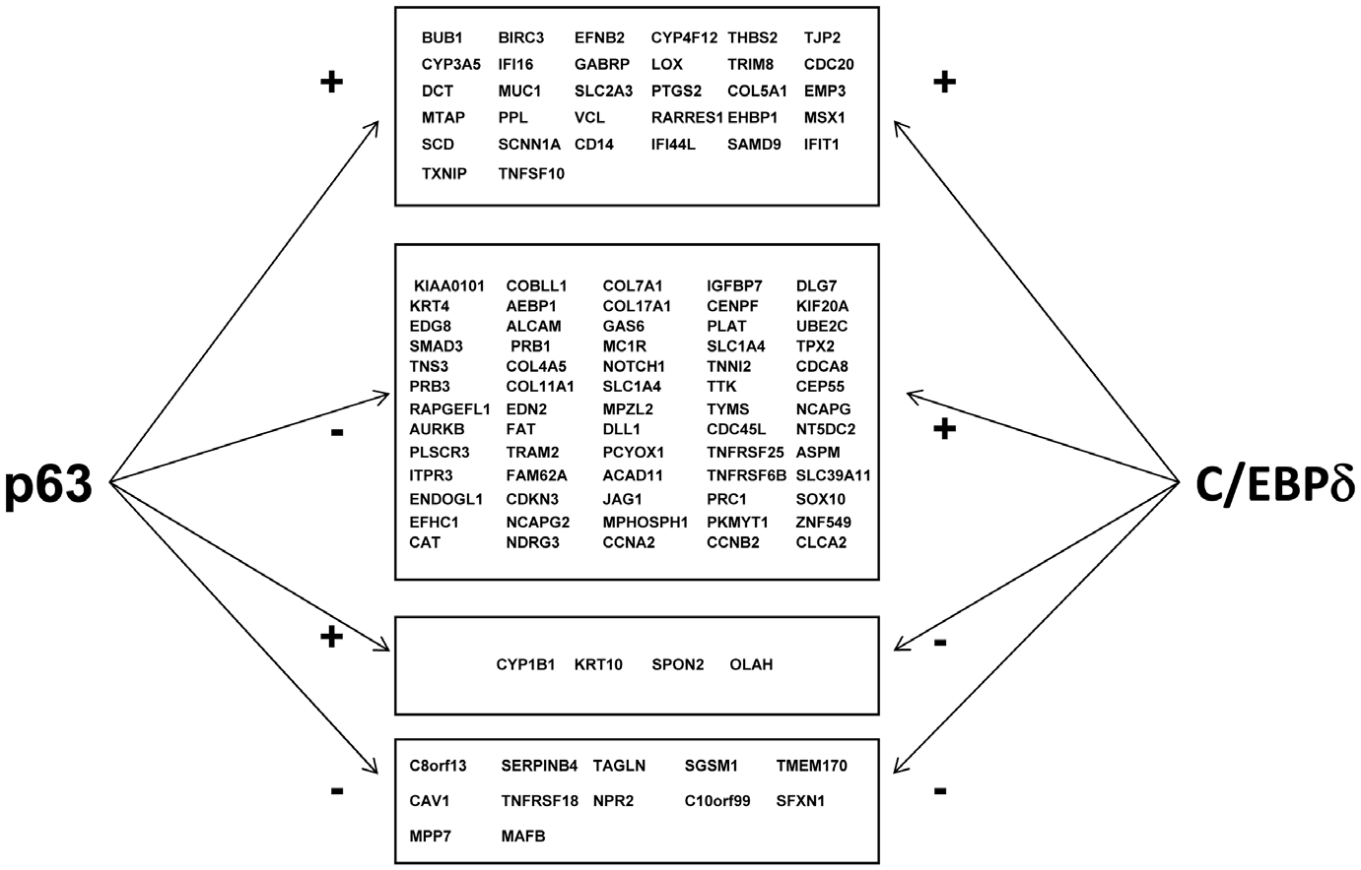
C/EBPδ and p63 regulated genes. Genes coregulated by p63 and C/EBPδ, either activated or repressed (Table S2). The data on p63 were derived from References 32, 36–39.

We have recently shown that some of the targets of p63 are also influenced by inactivation or overexpression of C/EBPδ [21]. We were therefore interested in verifying the overlap between the p63 targets and the C/EBPδ targets identified here. For this analysis, shown in Figure 4, we considered collectively the targets identified in various p63 profiling experiments performed with siRNA [32], [36]–[39]. The coregulated genes are grouped according to the p63 and C/EBPδ effects on them: the larger cohort is represented by genes that are repressed by p63 and activated by C/EBPδ, with only three genes behaving in the opposite way. Interestingly, several genes are either repressed or activated by both TFs. These data are in agreement with a different role of the two TFs in keratinocyte differentiation and with the overlapping expression patterns of p63 and C/EBPδ in human interfollicular epidermis [21].

### C/EBPδ and keratinocyte differentiation

**Figure 5:**
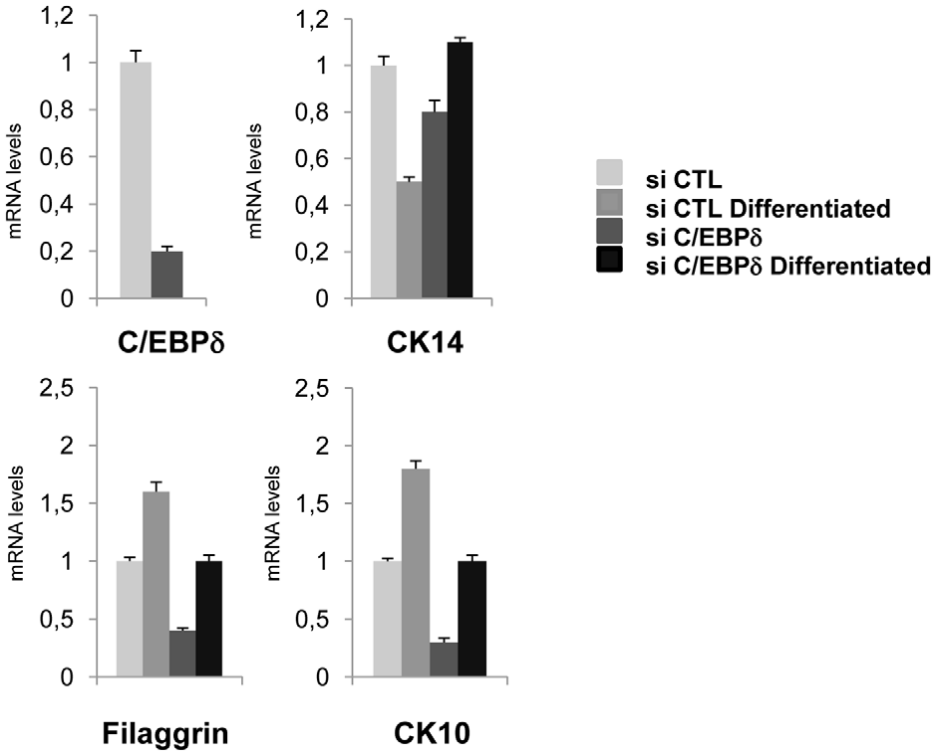
C/EBPδ is important for keratinocyte differentiation. Analysis of differentiation markers by qRT-PCR of C/EBPδ-inactivated cells induced to differentiate. Primary keratinocytes were transfected with scamble and C/EBPδ siRNAs. After 24 h, half of the cells were induced to differentiate by addition of calcium. Samples were harvested after 3 days, RNAs prepared and qPCR performed on the indicated genes. Values are normalized to GAPDH, used as an internal control.

We analyzed the specific role of C/EBPδ in keratinocyte differentiation by transfecting primary keratinocytes with C/EBPδ and scramble siRNAs and inducing differentiation *in vitro*; we compared the mRNA levels of differentiation markers in silenced and control cells. As shown in Fig. 5, qRT-PCR analysis reveals a substantial increase of the mRNA levels of filaggrin and KRT10 upon differentiation of control cells, but not in cells inactivated for C/EBPδ. On the other hand, the levels of KRT14 modestly decrease after differentiation in control cells, but not in siRNA-treated cells, suggesting that C/EBPδ indeed affects the expression of genes that mark keratinocytes differentiation.

### Role of C/EBPδ targets Sox2 and MafB, in keratinocyte differentiation

**Figure 6:**
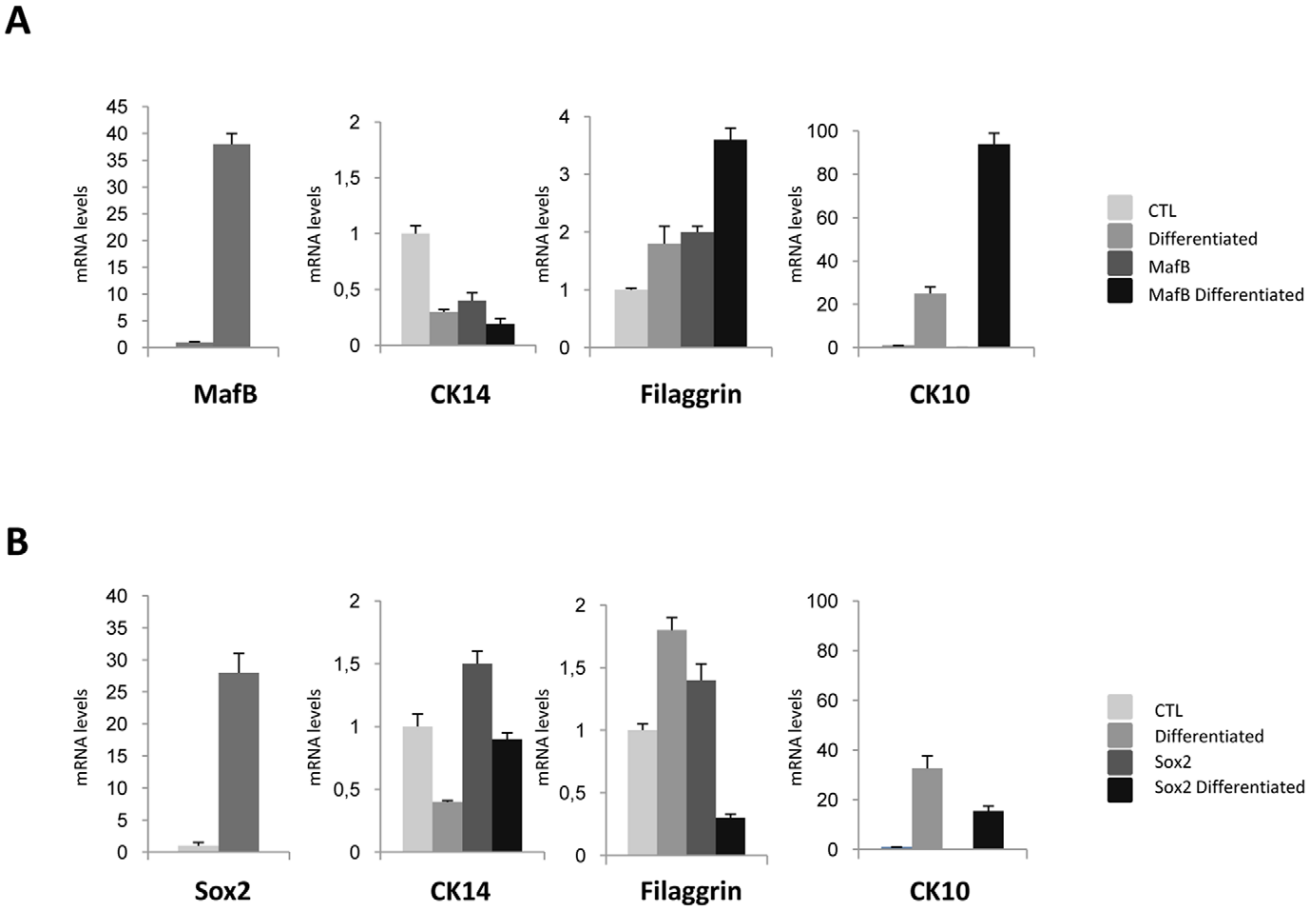
Role of C/EBPδ target genes in keratinocyte differentiation. **A**. Analysis of differentiation markers by qRT-PCR of primary keratinocytes induced to differentiate after transient transfection of MafB expression plasmid and empty vector as control. After 24 h, half of the cells were induced to differentiate by addition of calcium, as in Fig. 5. **B**. Same as A, except that Sox2 was overexpressed.

We further analyzed two C/EBPδ target genes -Sox2 and MafB- to verify their function in keratinocytes differentiation. Sox2 is one of the genes that leads to reprogramming of differentiated cells to pluripotent ES-like cells [40]. MafB is a TF with a role in the development and differentiation of several tissues [41]. The former is repressed by C/EBPδ, the latter activated. We transiently transfected primary keratinocytes with Sox2 and MafB expression vectors, evaluating by qRT-PCR the levels of endogenous markers of keratinocytes differentiation (Fig. 6): Sox2 caused a decrease of filaggrin and KRT10, whereas MafB overexpression resulted in higher levels of filaggrin and KRT10. The changes in the basal layer marker KRT14 were modest. These data suggest the levels of two TFs regulated by C/EBPδ impact on the expression of markers of differentiation.

### C/EBPδ is differentially expressed in human skin tumors

**Figure 7:**
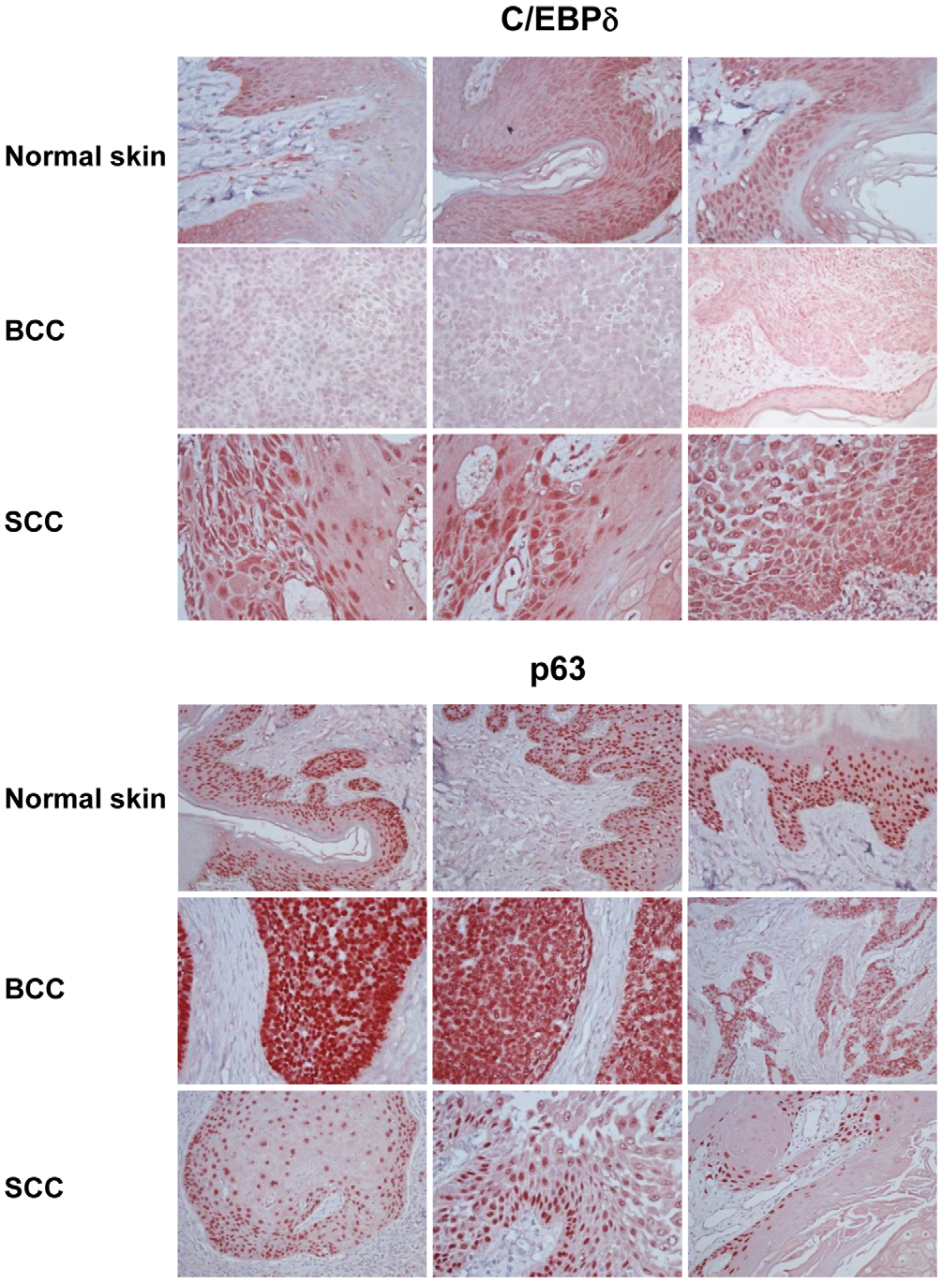
Overexpression of C/EBPδ in human non-melanoma skin tumors. Immunohistochemistry analysis of p63 and C/EBPδ in normal skin (Upper Panel), BCC (Middle Panel) and SCC (Lower Panel).

The expression of C/EBPα and β is altered in several tumors, including those of epithelial origin [23]–[27]. Nothing is known about the expression of C/EBPδ in various types of skin tumors. We decided to analyze its expression in a panel of different skin tumors by immunohistochemistry (IHC); the results are shown in Figure S2: 0 is no expression, and 3 is maximum positivity. Most Squamous Cell Carcinomas -SCC- show overexpression of C/EBPδ (14 out of 20); as expected, p63 levels were also generally high [42]. On the other hand, Basal Cell Carcinomas were negative for C/EBPδ expression, whereas p63 expression was very high. Figure 7 shows the IHC staining of representative cases of SCC and BCC, and comparison with normal skin. In conclusion, these data indicate that, unlike p63, C/EBPδ is differentially expressed in human BCC and SCC tumors and, like the related C/EBPβ, overexpressed in SCC.

## Discussion

The modulation of gene expression in growth and differentiation is controlled by transcription factors. In tissues in which these processes are continuously active to provide the required mass of specialized cells, many TFs contribute, with distinct yet partially overlapping programs. Hierarchical models have been suggested that specific TFs account for differentiation programs in a multistep way. This matter is complicated by the fact that TFs come in families, with members often being apparently redundant. In multilayered epithelia, it is clear that p63 plays a role that cannot be vicariate by other members of the family, p53 or p73. The recent identification of p63 targets provided links with other TFs that play an important role in skin biology, notably C/EBPs [21], [35].

### C/EBPδ and keratinocyte differentiation

C/EBPδ belongs to a family of TFs important for several differentiation pathways, including adipocytes, liver and haematopoietic lineages [2]. C/EBPδ and C/EBPβ are structurally the two closest members and genetic experiments have linked them to the early phases of adipocyte differentiation, with redundant roles [6]. Neither C/EBPβ nor C/EBPδ KO mice show a skin phenotype, possibly because of redundancy. Double C/EBPα/C/EBPβ KO have profound skin defects, with lack of complete differentiation and increased proliferation of basal layer cells, associated to increased p63 expression [18]. Among the established targets of C/EBPs are desmocollins, which are required for skin desmosome formation. Desmocollin 3, which is expressed in basal cells, is transactivated by C/EBPδ and C/EBPβ, the suprabasal Desmocollin 1 by C/EBPδ and C/EBPα [43]. In humans, little variation of expression of C/EBPδ was detected in the different phases of maturation of skin annexes, the hair follicle and the sebaceous gland, unlike other members of the family [44]. However, we observed that C/EBPδ staining is not uniform in human interfollicular skin. Co-expression with the growth promoting p63 is seen only in selected basal cells, it is balanced in the spinous layer, and in the upper granular layer, C/EBPδ predominates [21]. Among the many genes targeted by C/EBPδ and p63, the vast majority is controlled in an opposite way by the two TFs, which is consistent with their overlapping expression patterns in the skin.

**Figure 8:**
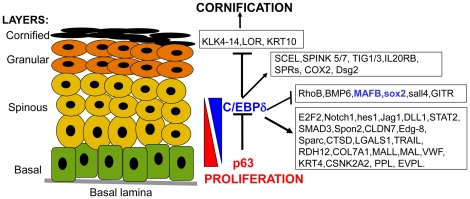
Scheme of C/EBPδ targets in human skin.

Many genes activated by C/EBPδ in our experiments are involved in functions of the granular layer and in the formation of the skin barrier (Fig. 8). SPRRs, RARRES3 and COX-2 are expressed in the granular layer and involved in differentiation [44]–[49]. KRT4 is a developmentally regulated Keratin, required for proper differentiation of internal multi-layered epithelia (50). Spink proteins are serine protease inhibitors involved in skin barrier formation: mutations in the Spink5 –LEKTI- gene in humans cause the Netherton syndrome, an ichthyosiform dermatosis (51); Spink5 KO mice have severe alteration in the Stratum Corneum (SC) [52]–[54]. Spink targets, such as KLK4, a serine protease implicated in the desquamation, are repressed by C/EBPδ [55]. BMP6 is a signaling molecule that inhibits stem cell proliferation and triggers cell cycle exit from transit amplifying cells [56]. KRT10, Loricrin and Late Cornified Envelope group, LCE1F, LCE3A and LCE3C are markers of the suprabasal layers [57]. The experiments of Fig. 5 confirm that KRT10 expression is affected by C/EBPδ removal. In summary, C/EBPδ joins the list of other TFs involved in barrier formation, such as IRF6 [58], [59], KLF4 [60], Gata3 [61], [62] and HBP1 [63]. Each is likely to promote the expression of subsets of differentiation genes. Importantly, KLF4, Gata3 and HBP1 are directly controlled by p63 [62]–[64].

### C/EBPδ and control of proliferation

To induce terminal differentiation, proliferation must be stopped: C/EBPs are largely thought to be growth suppressors. Experiments performed in cultured cells *in vitro* as well as in mice *in vivo* have generally supported this idea. Mechanisms related to control of cell cycle progression, rather than induction of apoptosis or senescence, have been suggested. The mechanistic details are apparently multiple, and range from a direct role on CDKs, to activation of CDKI genes, to repression of growth stimulatory genes [65]. Comparatively less is known about C/EBPδ, but the available data fully support this idea: it is induced upon several environmental changes that lead to growth arrest in mammary and lung epithelial cells [66]. Overexpression leads to growth arrest in mammary and prostate epithelial cell lines [67]–[70]. It was therefore somewhat expected that cell cycle terms would emerge from our analysis on C/EBPδ targets. However, the strong emphasis on G2/M genes is novel and unexpected, genes involved in kinetokore function during mitosis, such as BUB1, Aurora Kinase B and Borealin-CDCA8, as part of the CPC complex, KNTC1-ROD, genes recruited by AurKB, CENPF, consensins/NCAPD2 [71]–[73], as well as Cyclin B2, Cyclin A, CDKN3-KAP, CDC20. These findings could explain the chromosomal instability in mammary cells upon removal of C/EBPδ [74].

The Notch, Wnt and TGFβ pathways are well represented -Notch1, Hes1, JAG1, SMAD3, BMP6, FRAT1, LTBP1, Sall4- as expected for a TF with a global outlook. Growth suppressor genes are activated by C/EBPδ in primary keratinocytes: EDG-8 [75], STAT2, a TF expressed in the granular layer of the skin [76], E2F2 and IRF1. Specifically, E2F2 functions as a tumor suppressor in the skin, since inactivation cooperates with transgenic expression of Myc to enhance tumor development. Hemizygous E2f2^+/−^ mice showed increased tumor incidence. E2F2 inactivation apparently modifies gene expression patterns of some Myc targets [77]. IRF-1, also a target of p63, together with another member of the family, IRF-3 [35], is expressed throughout human skin [78] and it is generally considered a growth suppressor [79]. Yet, IRF1 is overexpressed in profiling experiments of Squamous Cell Carcinomas, amid other IFN-activated signature genes [80].

Among the genes repressed by C/EBPδ, three genes, apparently not expressed in the skin, stand out because of their role in stem cells: Sox2, Sall4 and BCOR. Sox2 and Sall4 are TFs that reprogram differentiated cells, including keratinocytes, to Embryonic Stem cells-like iPS, [81], [82]. BCOR is a marker of mesenchimal stem cells [83]. We specifically tested Sox2 and indeed overexpression has negative consequences on the expression of markers of terminal differentiation, Filaggrin and KRT10. An opposite result was obtained with MafB. This latter TF was more efficient in repressing the levels of the basal marker KRT14 before and after differentiation. MafB is a member of the large leucine zipper AP1 family, and specifically of the MAF subfamily [41]. MafB, as well as c-Maf, are highly expressed in basal keratinocytes and in the hair follicle at the late embryo stage, dropping after birth [84]. Genetic data generated by analysis of KO mice of other MAFs –Nrf2, MafG, MafK and the negative regulator Keap1- indicate that several members play a role in multilayered epithelia, notably of the esophagus [85].

C/EBPs expression is altered in many tumors, notably of epithelial origin [23]–[27]. In general, lack of expression has been reported which, in the case of C/EBPδ in cervical carcinomas, is caused by epigenetic silencing due to both DNA methylation of CpG islands and PcG-mediated deposition of negative histone marks [86]. Our analysis of non-melanoma skin cancers show that BCC are negative, but the majority of SCCs have relatively high levels of C/EBPδ, especially in areas of ongoing differentiation. Indeed, oncogenes, such as FRAT1, MALT1 and the TFs HOXB4, Sall4 and PTTG1 [87]–[89] are among the targets. KLF4 is another example of a TF with strong growth suppressive and pro-differentiation activities, that is nevertheless associated to tumor promoting functions in specific contexts. Our findings suggest that C/EBPδ could play different roles, depending upon the tissue context. More generally, it could serve a dual role in skin homeostasis, both in very early and late stages. In the former, it could limit, through its suppressive properties, the growth potential of early progenitors. In the later stages, it may coordinate cell-cycle exit and induce differentiation markers.

## Supporting Information

Figure S1 GO analysis of the C/EBPδ regulated genes identified by ChIP on chip.
(2.36 MB TIF)

Figure S2 Skin cancer tissue array results obteined by IHC.
(2.98 MB TIF)

Table S1 RT-PCR primers list.
(0.06 MB DOC)

Table S2 List of the primers used in ChIP assay.
(0.04 MB DOC)

Table S3 List of the C/EBPδ target genes identified by ChIP on chip analysis.
(0.04 MB XLS)

Table S4 List of the C/EBPδ target genes identified by expression profiling analysis.
(0.06 MB XLS)
