# Supplementary figures and images for: C/EBPδ Gene Targets in Human Keratinocytes
Source: PLoS One. 2010 Nov 2;5(11):e13789. doi: 10.1371/journal.pone.0013789 (PMC2970548; doi:10.1371/journal.pone.0013789)

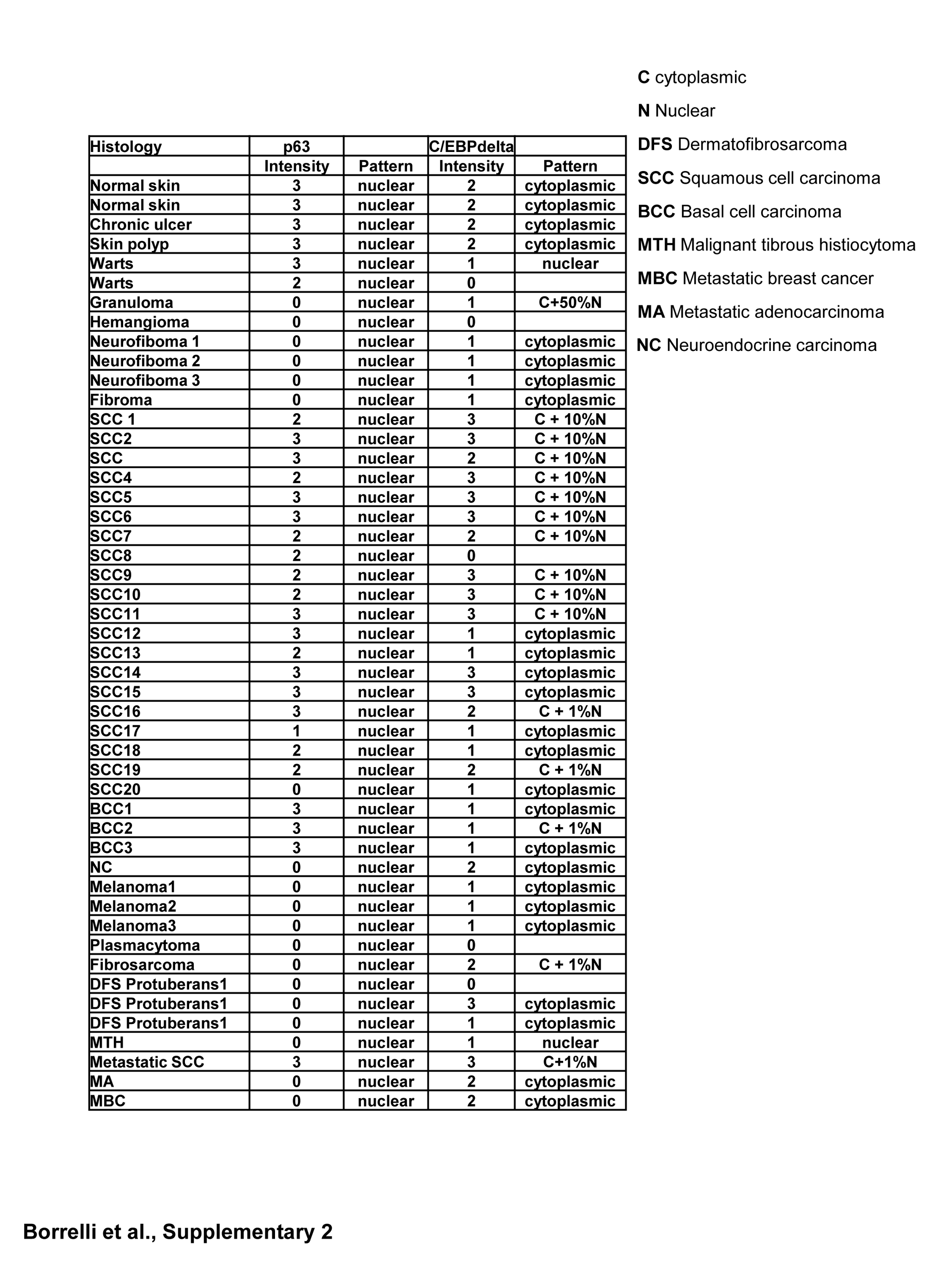

Supplement: Figure S1 — GO analysis of the C/EBPδ regulated genes identified by ChIP on chip. (2.36 MB TIF) [file pone.0013789.s001.tif]

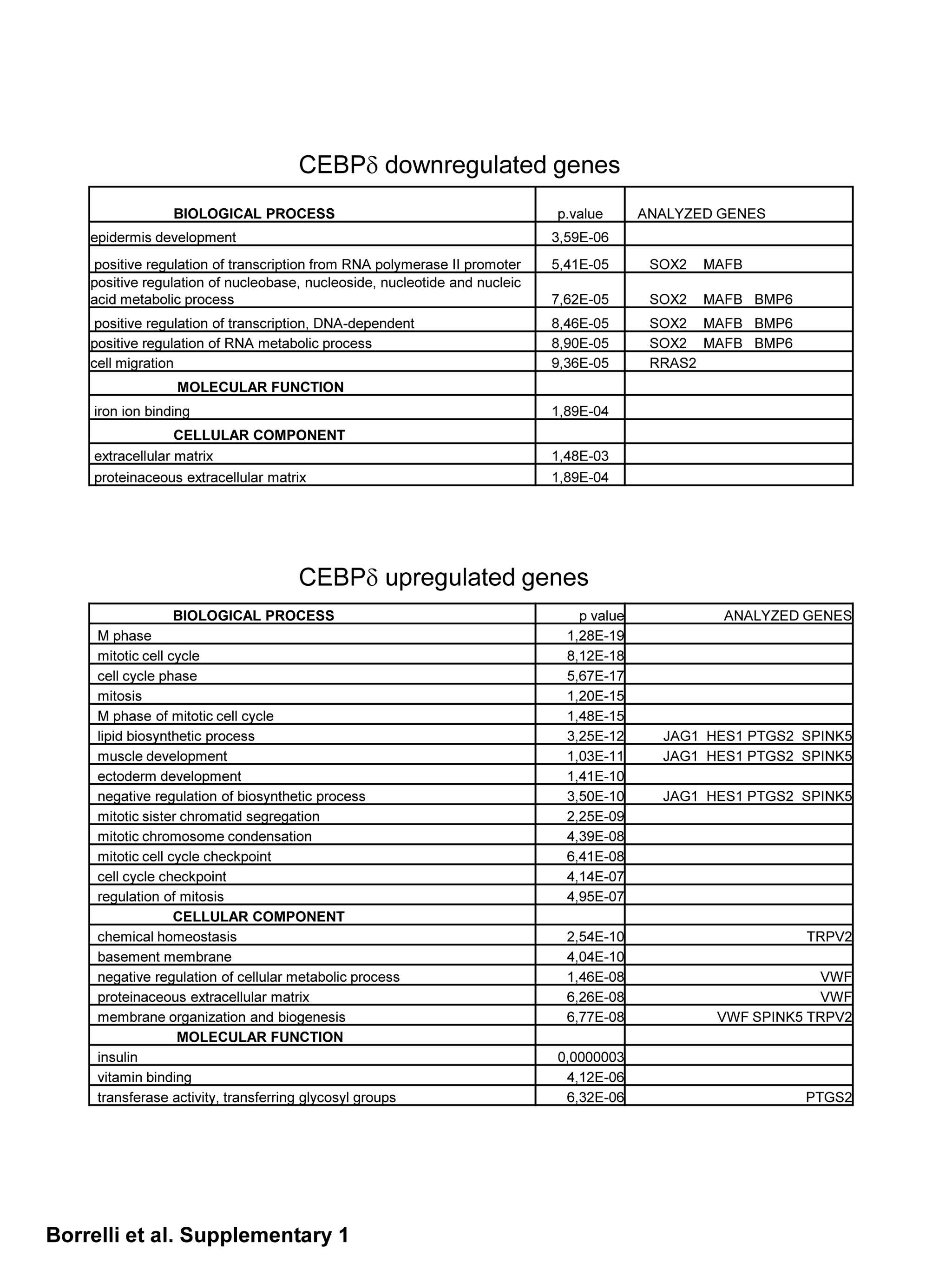

Supplement: Figure S2 — Skin cancer tissue array results obteined by IHC. (2.98 MB TIF) [file pone.0013789.s002.tif]
