# Supplementary material for: C/EBPδ Gene Targets in Human Keratinocytes
Source: PLoS One. 2010 Nov 2;5(11):e13789. doi: 10.1371/journal.pone.0013789 (PMC2970548; doi:10.1371/journal.pone.0013789)
Supplement: Table S1 — RT-PCR primers list. (0.06 MB DOC) [file pone.0013789.s003.doc]

| RT_VWF_for | TCGTCAACACCTTTGATGGGAGCA |
| --- | --- |
| RT_VWF_rev | AGAGGCTCACTCTCTTGCCATTCT |
| RT_TNFRSF14_rev | TACATTGCCCACCTCAATGGCCTA |
| RT_TNFRSF14_for | TTCTCTGTCCTGGAGCAGTTCCG |
| RT_TNFRSF25_for | AACCACGACGGGCAGAGAGCA |
| RT_TNFRSF25_rev | ATCTTCTTGTGGAAGTCACCGGCA |
| RT_TOP2A_for | ACCATGGAAGTGTCACCATTGCAG |
| RT_TOP2A_rev | CTGGGTCACTAATTCCACAGAACC |
| RT_TEGT_for | AGTCAGAGCACATCCGGTGTTAGA |
| RT_TEGT_rev | ATGTTCATGGTTCCAGAGTCCGTG |
| RT_STAT2_for | TCAGGATCAGCTGCACCAGCTTTA |
| RT_STAT2_rev | TGCAACGGCCACACTCATAGTTCA |
| RT_SOX2_for | TCAGGAGTTGTCAAGGCAGAGAAG |
| RT_SOX2_rev | GCCGCCGCCGATGATTGTTATTAT |
| RT_SALL4_for | GGCCAATAGTCAAGCCGAAAGCAT |
| RT_SALL4_rev | CCCACAAATGTGCCAGGAACTTCA |
| RT_MAFB_for | AAGAGAGCTAGAGAGCGAGCAACG |
| RT_MAFB_rev | CCGGCCAAGCCTTTGTCT |
| RT_MAL_for | TGCCCGACTTGCTCTTCATCTTTG |
| RT_MAL_rev | TCAGCTCAAGTTCTACTGCGGCTT |
| RT_LZTS_for | TACGAGAGGGAGAAGACCAGCTT |
| RT_LZTS_rev | TGTCCTTCAGCTGTGCCTTGAGA |
| RT_LGALS1_for | ATCTCTCTCGGGTGGAGTCTTCTGA |
| RT_LGALS1_rev | GTTTGAGATTCAGGTTGCTGGCGA |
| RT_JAG1_for | AACACCTTCAACCTCAAGGCCA |
| RT_JAG1_rev | TGTCATTACTGGAATCCCACGCCT |
| RT_HES1_for | GTCAACACGACACCGGATAAACCA |
| RT_HES1_rev | TCAGCTGGCTCAGACTTTCA |
| RT_TNFAIP8L1_for | GGTTGGACGTACGGACTCTG |
| RT_TNFAIP8L1_rev | AGCACCTCACTGCTGGTGTC |
| RT_TNFRSF18_for | ATGTGTGTCCAGCCTGAATTCCAC |
| RT_TNFRSF18_rev | CACAGTCGATACACTGGAAGCCAA |
| RT_TIG1_for | AGTTCACGTGGTCTTCAGCACAGA |
| RT_TIG1_rev | ACACTCGAGCAGAACATTTCCC |
| RT_BMP6_for | CGGACATGGTCATGAGCTTTGTGA |
| RT_BMP6_rev | AACTCTTTGTGGTGTCGCTGACGA |
| RT_KRT10_for | AAAGCATGGCAACTCACATCAGGG |
| RT_KRT10_rev | TGGCATTGTCGATCTGAAGCAGGA |
| RT_RRAS2_for | TGTGTGATAGATGACAGAGCAGCC |
| RT_RRAS2_rev | AGCCTTCGCCAGTCCTCATATACT |
| RT_MREG_for | TCGTAATCAGCAGGCCAAAGACTC |
| RT_MREG_rev | TTCTTACTTCCCTTCGAACCTGCC |
| RT_TRPV5_for | TGCTACTGAGAAGCTCCGGGAT |
| RT_TRPV5_rev | TGTGCAGATGGAGATGTCAAGGGT |
| RT_KRT4_for | GCAGCTAGATACCTTGGGCAATGA |
| RT_KRT4_rev | AGGACCACAAAGTCATTCTCGGCT |
| RT_COX2_for | TTCCATTGACCAGAGCAGGCAGAT |
| RT_COX2_rev | GCATCGATGTCACCATAGAGTGCT |
| RT_TRAIL_for | AACTGGGACCAGAGGAAGAAGCAA |
| RT_TRAIL_rev | ATGCCCACTCCTTGATGATTCCCA |
| RT-E2F2_for2 | TCCGCAGACGAGACTGGAAGTG |
| RT-E2F2_rev2 | TCTGGGCACAGGTAGACTTCGAT |
| RT_RARRES3_for2 | TGCGAAGGAGATGGTTGGTCAGAA |
| RT_RARRES3_rev2 | CACACCAACTTCAACCTTGGCCTT |
| RT_TRPM4_for | ATGCCTACGGAGAGCTGGACTTCA |
| RT_TRPM4_rev | CCCATGTGCGTGTGACCAGACTATAA |
| RT_SPINK5_for | TCACAGAAGAGGGCCAGGCATTTA |
| RT_SPINK5_rev | TTTCCCATCTGTGCCACAAACAGC |
| RT-TNFRSF6B_for2 | AGAGCGTCATGCAGGTCTTCTG |
| RT-TNFRSF6B_rev2 | TAGGTGGGTGTTTCTGCCACT |
| RT_RDH12_for | AGTTGGAACGATGCTGGTCACCTT |
| RT_RDH12_rev | GCCAGGAAGCTGCACATTTGTTCT |

Borrelli et al. Supplementary table I
