# Supplementary material for: C/EBPδ Gene Targets in Human Keratinocytes
Source: PLoS One. 2010 Nov 2;5(11):e13789. doi: 10.1371/journal.pone.0013789 (PMC2970548; doi:10.1371/journal.pone.0013789)
Supplement: Table S2 — List of the primers used in ChIP assay. (0.04 MB DOC) [file pone.0013789.s004.doc]

| CDH12 for | ACCAAACTTAGCCAGAGACTGC |
| --- | --- |
| CDH12 rev | TGTCTCCCTACAGGAGGACTGT |
| CTNNAL1 for | CTGTCAACTTCGTAAGGGAAGG |
| CTNNAL1 rev | GATGAGCTGGGTACATCAACCT |
| CXCR4 for | GAGGAGAAACGACTGGAAAGAG |
| CXCR4 rev | ACCCAGTGTCAAGAACAGTCAC |
| HOXB4 for | CACACGAAGACTCCTTCCTCTT |
| HOXB4 rev | GAGGTGACCCACACAGACAATA |
| ING4 for | TCTCAAGGCCTGATACTCTTCC |
| ING4 rev | GGAGAACTTCCGCCCTTACTAT |
| LDLR for | AATCCTGCTGAGAGGTTAGCAC |
| LDLR rev | TTTCTCCCCTTAGGAGAAGGAG |
| PRMC1 for | GTTAATGCCTGGTGACTGAGC |
| PRMC1 rev | TGTTAGAGCCCTACACGACTCA |
| TBRG1 for | TTTCCTATCTCCTCCCTCCTTC |
| TBRG1 rev | CACTGAGCAACTCATTCAGTCC |
| TRPM2 for | GAATCACTTGAACCAGGGAGAC |
| TRPM2 rev | GTACAATTCTGCACAGCTCCTG |
| GATA3 for | CCTAATAACGGGAGACCAAGTG |
| GATA3 rev | CAACTTCACCTTGGTGTGTGAG |
| TSLP for | GATACCCTGCCTACCTCATCTG |
| TSLP rev | ACTAGAACCTTCGGCTTCAT |
| MAFB for | TTGGGTCTGAACACAACTCTCGGA |
| MAFB rev | TCGTGCGTTCCTGTTTCTGGAGAT |
| LTBP1 for | GACGGGAAAGAGTAGAACAAGG |
| LTBP1 rev | GAACACAGTTTGAGAGGCACTG |
| BCOR for | GGTACAGACCGTGAAAATGCTC |
| BCOR rev | CTCAGCCAAAGCTGTCAAACC |
| LBR for | GTAGCTGGGACTACAGGTGCTC |
| LBR rev | TGGGGACCTAAGACAGATCCTA |
| NF2 for | CTACACCCGTTAGAACCCTTGA |
| NF2 rev | ACCTACCTCGTGAGGTTGTTGA |

Borrelli et al. Supplementary table II
